# Asymmetry of nanoparticle inheritance upon cell division: Effect on the coefficient of variation

**DOI:** 10.1371/journal.pone.0242547

**Published:** 2020-11-17

**Authors:** Tim Lijster, Christoffer Åberg

**Affiliations:** Groningen Research Institute of Pharmacy, University of Groningen, Groningen, The Netherlands; University of Edinburgh, UNITED KINGDOM

## Abstract

Several previous studies have shown that when a cell that has taken up nanoparticles divides, the nanoparticles are inherited by the two daughter cells in an asymmetrical fashion, with one daughter cell receiving more nanoparticles than the other. This interesting observation is typically demonstrated either indirectly using mathematical modelling of high-throughput experimental data or more directly by imaging individual cells as they divide. Here we suggest that measurements of the coefficient of variation (standard deviation over mean) of the number of nanoparticles per cell over the cell population is another means of assessing the degree of asymmetry. Using simulations of an evolving cell population, we show that the coefficient of variation is sensitive to the degree of asymmetry and note its characteristic evolution in time. As the coefficient of variation is readily measurable using high-throughput techniques, this should allow a more rapid experimental assessment of the degree of asymmetry.

## Introduction

How nanoparticles interact with cells is important for many applications of nanotechnology, most directly when nano-sized objects are being used to diagnose and treat disease [1–7], but also indirectly when nanoparticles released from products containing nanoscale features may subsequently interact with biology [8–11]. While *in vivo* studies remain the gold standard, nanoparticle-cell interactions are nevertheless often investigated *in vitro* and commonly using cancerous cell lines to allow for more rapid experimentation. Cancer cells generally divide rapidly (*e*.*g*., the cell population doubling time of HeLa cells is typically less than a day) implying that cell divisions are, implicitly or explicitly, a common feature of many *in vitro* experiments.

In this context, it has repeatedly been observed that when cells divide, the nanoparticles they have taken up are shared between the resulting daughter cells in an asymmetrical fashion [12–22], that is, one daughter cell receiving more of the nanoparticles from the mother than the other daughter cell. Evidence for such an asymmetry includes fits of computational [13,14] and theoretical [15,16,20] models to experimental data, as well as more direct observations by tediously imaging individual cells as they divide and subsequently tracking the nanoparticle inheritance pattern of the daughter cells [19–22].

Here we complement these approaches by showing that the coefficient of variation over the cell population (*i*.*e*., the standard deviation over the mean) is a useful observable to quickly assess the degree of asymmetry upon cell division. We have previously developed a model of nanoparticle uptake in dividing cell populations and demonstrated that it describes experimental observations well [23–26]. With this firm basis, we perform simulations of the evolution of the cell population and, specifically assess how the coefficient of variation evolves in time both after a nanoparticle exposure as well as during continuous exposure. While an increase of the coefficient of variation with time after exposure has been observed previously [13,20], we here show that such an increase is characteristic of asymmetric inheritance; for symmetric inheritance, the coefficient of variation instead oscillates. Furthermore, between these two extremes of symmetry and asymmetry, a mixed behaviour is observed. Thus, how the coefficient of variation evolves in time after exposure is sensitive to the degree of asymmetry. Measurement of the coefficient of variation is far easier than a full assessment, and modelling, of the whole distribution. Consequently, we suggest that its time evolution allows a ready assessment of potential asymmetry in nanoparticle inheritance upon cell division for future studies.

## Results and discussion

From the outset, it is important to distinguish between two different notions of asymmetric inheritance upon cell division. The first notion is befitting when discussing what happens at cell level. Thus, consider a cell that has 100 nanoparticles and when it divides, the two daughter cells take, say, 43 and 57 of those nanoparticles, respectively. Clearly we may consider this particular division as asymmetric.

However, we may also consider the same process at the particle level. Thus, say that each of those 100 particles has an equal probability of ending up in daughter cell 1 or 2. The most probable inheritance is then, indeed, that both daughter cells take 50 nanoparticles. Nevertheless, it will often be the case that the daughter cells take something close to 50, but not exactly 50. For example, the probability of one daughter inheriting 43 and the other 57 nanoparticles is 0.03, while the probability of both receiving 50 nanoparticles is 0.08 (see below for how to determine this). Certainly, the latter is a more probable event, but if we consider also the probability of one daughter receiving 49, 48, 47 *etc* nanoparticles, then it is clear that most of the time, the inheritance at a cell level is asymmetric, at least to some degree. In essence, the reason for this is simply the assumption that the inheritance is stochastic at a single-particle level. In other words, regardless of the fact that the inheritance is completely symmetric for a *single particle*, at the *cell level* it is typically asymmetric.

Of course, there is the possibility that even at a single particle level, the probability that a particle is inherited by a certain daughter cell is skewed towards one of the daughter cells, so that the probability of daughter cell 1 inheriting a nanoparticle is *p*, while the probability of daughter cell 2 receiving the nanoparticle is 1—*p*. Here *p* must be between 0.5 (completely symmetric) to 1 (completely asymmetric).

The initial report [15] on asymmetric inheritance of nanoparticles explicitly considered the asymmetry at single-particle level and, indeed, reported a probability, *p*, between 0.52 and 0.72 [15]. Other workers have sometimes been a bit less explicit when it comes to their definition of asymmetry. In light of the fact that asymmetric inheritance at a cell level is a trivial consequence if the inheritance is stochastic, we consider that the more interesting question is the degree of asymmetry at single-particle level. Hence, in the following we will stick to the original definition of asymmetry.

Thus, in order to model the inheritance upon cell division, we consider a cell containing *n* nanoparticles which divides into two daughter cells and where the probability of daughter cell 1 inheriting a nanoparticle upon cell division is *p*. It is well-known that the probability of daughter cell 1 inheriting *k* nanoparticles is then given by a binomial distribution [15]. Since which daughter cell is number 1 or 2 is arbitrary, the overall inheritance distribution is given by a sum of binomials, *viz*.

$$\frac{1}{2}\left(\begin{array}{c} n\\ k \end{array}\right)(p^{k}{\left(1-p\right)}^{n-k}+p^{n-k}{\left(1-p\right)}^{k}).$$ (1)

**Fig 1:**
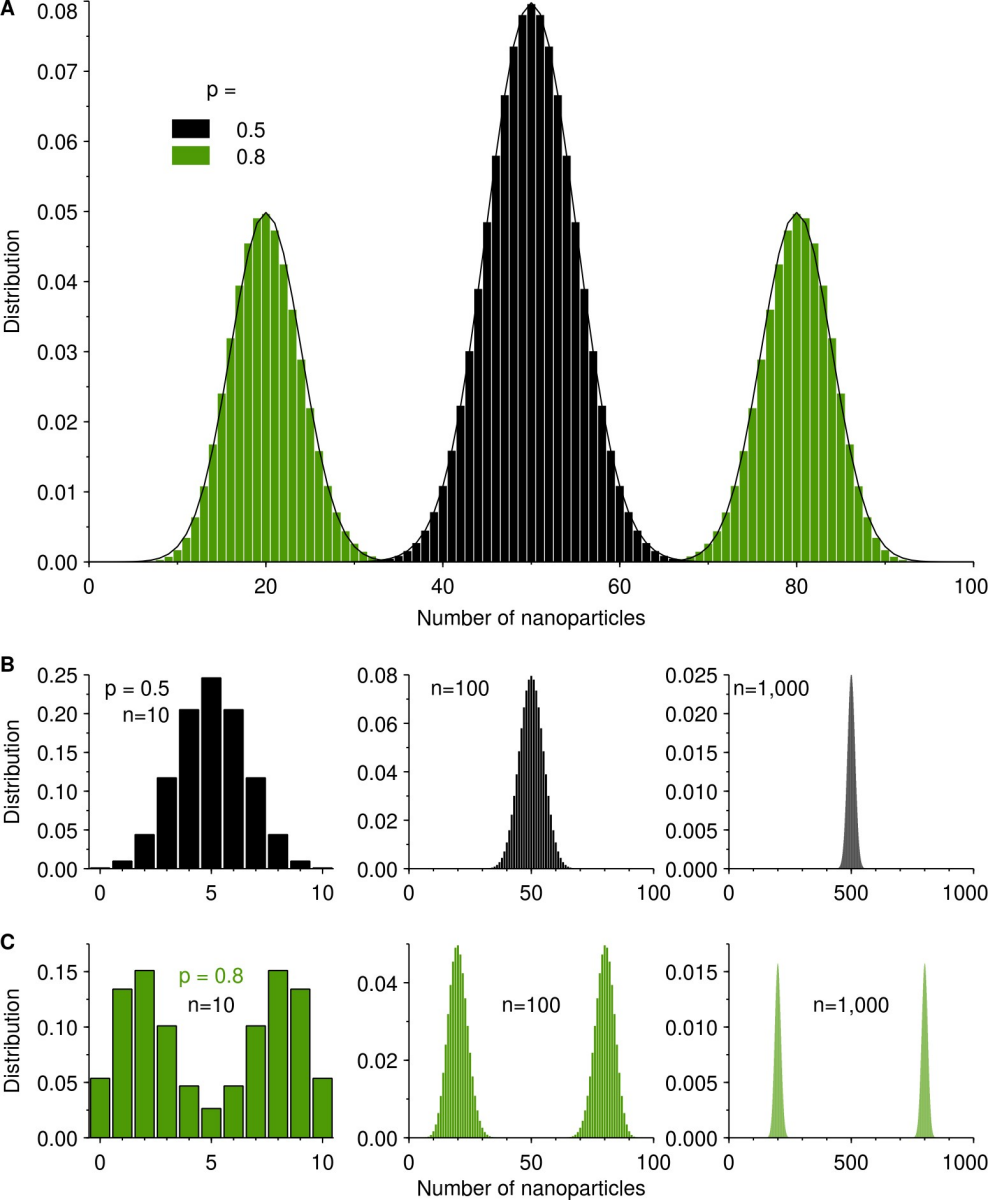
Inheritance distribution [Eq (1)]. **A.** Inheritance distribution for a cell containing 100 nanoparticles upon division, for symmetric inheritance [*p* = 0.5 in Eq (1)] and highly asymmetric inheritance (*p* = 0.8) as indicated by the legend. (Solid lines) Normal distribution approximation (see Methods). **B-C.** Inheritance distribution for different number of particles (n = 10, 100 and 1000 as indicated in figures). **B.** Symmetric inheritance [*p* = 0.5 in Eq (1)]; **C.** Highly asymmetric inheritance (*p* = 0.8).

We show this distribution for a cell containing 100 nanoparticles in Fig 1A, both for a completely symmetric inheritance [*p* = 0.5 in Eq (1)] as well as for a highly asymmetric inheritance (*p* = 0.8). These results illustrate more vividly the discussion above: Even for a completely symmetric inheritance at single-particle level, the distribution (Fig 1A; blue) nevertheless has a width and most of the time, a recently divided cell will not take half of the nanoparticles of the mother. For asymmetric inheritance at single-particle level, the distribution (Fig 1A; red) splits into two peaks and essentially all divisions will be asymmetric at cell level.

It is noteworthy that the inheritance distribution is highly dependent on the number of nanoparticles, as illustrated in Fig 1B and 1C which shows the inheritance distribution for 10, 100 and 1000 particles and for two different asymmetries. This requires careful consideration when comparing to data stemming from experimental techniques that do not directly measure the number of nanoparticles, but proxies thereof (*e*.*g*., fluorescence intensity [27]). For such cases, the actual number of nanoparticles is obviously not known. However, as will transpire, the results we present below are only weakly dependent on actual numbers, at least when the numbers concerned are 100 or more particles, so this seems not to be an issue in practice. Whether it could actually be used as a basis for a method to convert from, say, fluorescence to number of nanoparticles is an interesting idea, but one that we do not pursue further here.

From Fig 1B and 1C we also conclude that if a cell has fewer nanoparticles before division, then the inheritance distribution can become quite wide, relatively speaking. This is particularly so for larger asymmetries at single-particle level [larger *p* in Eq (1)], leading to overlapping of the two peaks; conversely, for larger number of nanoparticles, the peaks become, relatively speaking, more narrow and well-separated. This is not a novel phenomenon, but rather is related to small-number fluctuations. However, it can have very practical implications. Thus, Braeckmans, De Smedt and colleagues suggested that nanoparticles delivered into cells within vesicles are unsuitable for long-term tracking of cells due to asymmetry upon cell division, while if they are delivered into the cytosol, the inheritance is symmetric and tracking can be performed for longer [20]. While the symmetrical inheritance is a prerequisite, Fig 1B shows that it is not sufficient to ensure a symmetrical inheritance at cell level; a large number of labels is also required (*cf*. the results for *n* = 100 and 1000 in Fig 1B)–undoubtedly an interesting application, or rather avoidance of, small-number fluctuations. More generally, cell division is known to be a cause of variability also for organelles and biomolecules (*e*.*g*., proteins and RNA molecules), especially when the number of objects is low [28].

We end this general discussion about asymmetrical inheritance with the further qualification that we should actually not consider the particles themselves, but what happens to the nanoparticle-containing organelles [15,18,29], as presumably it is the organelles that are being inherited by daughter cells. The distinction arises because some nanoparticles may reside within the same organelle and would presumably be inherited as a unit. We will avoid this complication in the following, which would be reasonable if organelles are mostly similarly populated, including due to nanoparticles not agglomerating in suspension and being taken up as single particles. Suffice to say here, that it is possible to take into account a differing number of nanoparticles in the organelles by deconvolving the number of nanoparticle-containing organelles with the organelle occupancy distribution (or organelle fluorescence distribution in the case of methods based on fluorescence) and this has been described previously [29].

Given the interest in asymmetric nanoparticle inheritance, we posed the question whether there exist simple, high-throughput, experiments that could explicitly measure or at least give an indication of the degree of asymmetry even without detailed mathematical modelling [13–16,18,20] or tedious tracking of single cells [19–22]. We have previously argued, both from a theoretical perspective [24] as well as with simulations [23], that the mean nanoparticle uptake over a cell population is insensitive to (potential) asymmetries of the inheritance distribution. The reason is simple: when a cell divides, regardless of the asymmetry, the *mean* inheritance remains 50%. This line of thinking, however, raised the possibility that the standard deviation (the next order of statistical descriptors) of the number of nanoparticles per cell over a cell population could be sensitive enough to the inheritance distribution and thereby be used to demonstrate asymmetry. More precisely, we zoned in on the coefficient of variation (CV), that is, the standard deviation divided by the mean, as a normalised version of the width of the distribution.

Thus, we performed simple simulations of a continuously evolving cell population [23–26], where cells age as time goes on and, when reaching the end of one full cell cycle, cells divide into two new daughter cells that start the cell cycle anew. While the total duration of the cell cycle in reality is not the same for all cells, the variation is nevertheless moderate for cell lines, with a coefficient of variation of around 20% [30,31]. In our previous work on A549 cells we also showed good agreement between experiments and a parameter-free model that assumes the total duration of the cell cycle is the same for all cells [23,24]. For simplicity, we therefore assumed that all cells age at the same rate and have the same total duration of their cell cycle; however, below we also show results when this condition is relaxed. We also assumed that the cells were in the exponentially growing phase, which implies that the duration of one cell cycle is equal to the cell population doubling time. In order to make the discussion general, we present all our results with time normalised to the total duration of the cell cycle. For rapidly growing cells (*e*.*g*., HeLa) with a cell population doubling time of around a day, this implies that times can, roughly, be interpreted as days; for more slowly growing cells, the “actual time” may be found by multiplying our results with the cell population doubling time. We simulated experiments where cells took up nanoparticles continuously, as well as experiments where uptake for a limited duration (a “pulse”) was followed by a subsequent observation time (a “chase”). When cells divide, the nanoparticles they contain were shared between the daughter cells in accordance with the inheritance distribution [Eq (1)]. More details may be found in the Methods section and our previously published work [23–26].

**Fig 2:**
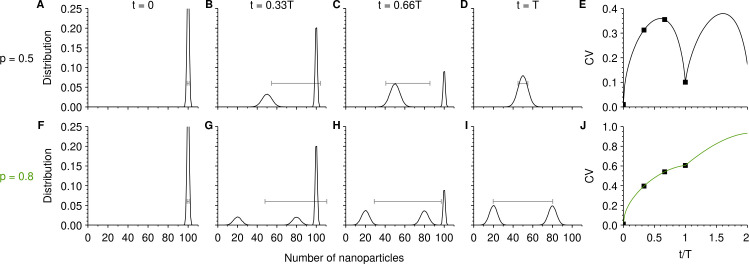
Time-evolution of the number of nanoparticles per cell due to cell division with a narrow initial distribution. Upon cell division, the nanoparticles taken up were shared between the daughter cells with a given inheritance distribution [Eq (1)]. The upper row is for symmetric inheritance [*p* = 0.5 in Eq (1)], while the lower is for a highly asymmetric inheritance (*p* = 0.8). **A-D** and **F-I.** Distribution of number of nanoparticles per cell, for the times indicated at the top. (Grey lines with bars) Mean ± standard deviation over the full population. The distribution is cut off in panels A and F, in order to keep panels A-D and F-J on the same scale. **E** and **J**, Time-evolution of the coefficient of variation (CV) under the two different conditions. The datapoints indicate the times for which panels A-D and F-I, respectively, show the distribution. The initial distribution was chosen as a normal distribution with a standard deviation of 1.

Before delving into more realistic circumstances, we discuss the case that the majority of cells of a cell population initially have roughly the same number of nanoparticles and subsequently evolve only due to cell division. In other words, we do not explicitly take into account the uptake process and we use a much more narrow initial distribution than one finds experimentally. This situation is sufficiently simple that it can be understood in detail and allows us to illustrate the general idea most clearly, before moving on to wider and more realistic initial distributions. Thus, consider Fig 2 which shows how the distribution of number of nanoparticles per cell evolves with time. We start by discussing the condition that the inheritance is symmetric [*p* = 0.5 in Eq (1); upper row of Fig 2]. Initially, most cells have 100 nanoparticles, but we include a limited spread around this value (Fig 2A). As time progresses, some cells will divide and thereby dilute their nanoparticle load. This results in a secondary peak which is centered on half the initial load, that is, on p⋅100 = 50 (Fig 2B). Naturally, cells that have not yet divided remain within the original peak, though their abundance progressively decreases with time (Fig 2B and 2C). After one full cell cycle, all cells have divided and only the secondary peak remains (Fig 2D). Since we assume that the inheritance is stochastic, the secondary peak is wider than the original, reflecting the width of the inheritance distribution (Fig 1A; blue).

Fig 2A–2D show also the consequences this evolution has on the mean and standard deviation of the number of nanoparticles per cell (line with bars in grey). Initially, the standard deviation is small (Fig 2A), reflecting the limited spread within the population of this simplified situation. As some cells divide, the standard deviation grows substantially (Fig 2B), because it now represents the standard deviation over two well-separated peaks. As time progresses, the standard deviation continues increasing as more and more cells divide, but then starts to decrease when the initial peak is no longer dominating the population (Fig 2C). After one full cycle, the standard deviation reaches a new minimum (Fig 2D), now reflecting the width of the secondary peak, which is wider than the original distribution, but nevertheless a single peak. A similar evolution may be observed for the coefficient of variation (Fig 2E) though its time-dependence is slightly more complex to consider in detail since it is the ratio between standard deviation and mean, both of which vary with time.

Consider now instead the situation that the inheritance is highly asymmetric [*p* = 0.8 in Eq (1)]; lower row of Fig 2) and assuming the same initial distribution (Fig 2F). In this case, when cells divide *two* secondary peaks are formed (Fig 2G), one centered on (1—*p*)⋅100 = 20 and one on *p*⋅100 = 80 nanoparticles. They represent, respectively, the cells that took the least number of nanoparticles upon cell division, and the ones that took the most. As time progresses, more and more cells divide and the original peak decreases and the two secondary ones grow (Fig 2G and 2H). After one full cell cycle, only the two secondary peaks remain, representing the inheritance distribution (Fig 1A; red) convoluted with the original distribution (Fig 2F).

The standard deviation of the distribution of number of nanoparticles per cell (line with bars in grey in Fig 2F–2I) shows a similar evolution in time as for a symmetric inheritance (Fig 2A–2D). The main difference is that for an asymmetric inheritance, the standard deviation is even more substantial, reflecting the larger width of the underlying inheritance distribution (Fig 1A; red). Furthermore, it remains large even after one full cell cycle (Fig 2I), again a reflection of the underlying inheritance distribution. Also in the case of a highly asymmetric inheritance, the standard deviation reaches a maximum before settling on a (new) minimum after one full cell cycle (Fig 2I; see also S1 Fig). However, when we consider the time evolution of the coefficient of variation, we also have to factor in how the mean evolves in time. Since, there is a simultaneous monotonic decrease of the mean, the net effect on the coefficient of variation is a monotonic increase with time (Fig 2J).

Considerations such as these give the following picture for how the coefficient of variation of the number of nanoparticles changes with time: There is an overall growth with each cell cycle and this growth is larger the more asymmetric the inheritance is (*cf*. the scale of Fig 2E and 2J). On top of this overall growth, there are potentially oscillations within each cell cycle. For a symmetric inheritance, the oscillations are prominent (Fig 2E), but the more asymmetric the inheritance, the less clear are the oscillations. For highly asymmetric inheritance, the oscillations disappear and the coefficient of variation grows monotonically (Fig 2J). Overall, it is thus clear that how the coefficient of variation evolves over a cell cycle is strongly dependent on the asymmetry of the inheritance.

As discussed above, the inheritance distribution is dependent on the actual number of nanoparticles, which could complicate the generality of the analysis. However, the time evolution of the coefficient of variation remains very similar, regardless if we assume that the number of nanoparticles is 100 (as in Fig 2), 1,000 or 10,000 (S2 Fig). In practice, the overall conclusions thus appear to be fairly general.

Furthermore, to simplify the picture we considered a very narrow distribution of the number of nanoparticles per cell in our discussion (Fig 2). In reality, the distribution is typically wide. For example, in our previous work on polystyrene nanoparticle uptake by A549 cells, we found that the experimental data was well-fitted by a log-normal distribution [23,24]. However, even with such a wide distribution, the time-evolution of the coefficient of variation shows, largely speaking, similar trends (S3 Fig).

**Fig 3:**
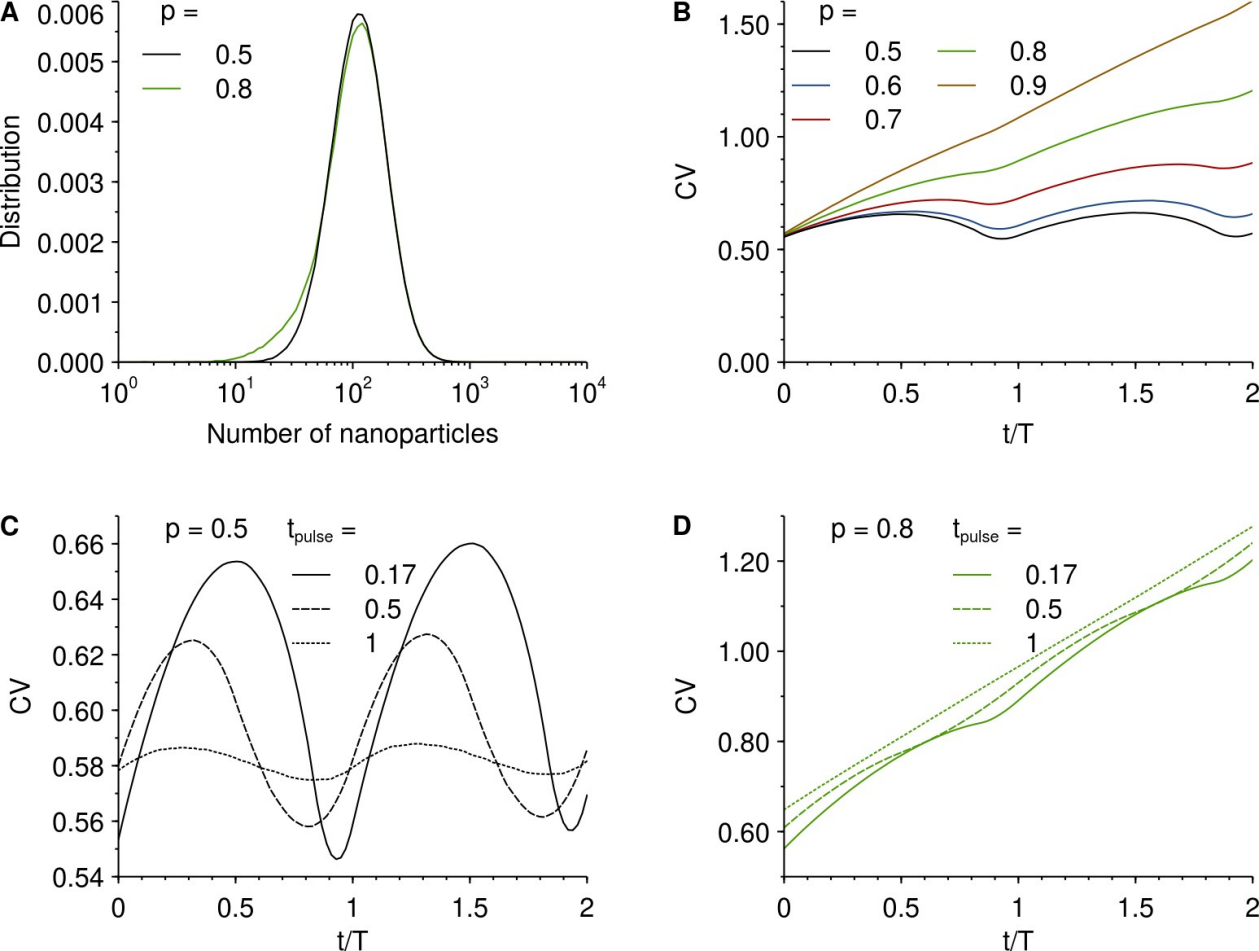
Time-evolution of the coefficient of variation of the number of nanoparticles per cell after a nanoparticle exposure. Cells were allowed to take up nanoparticles for a limited period of time (“pulse”) and then followed (“chased”). During the exposure, the cells took up nanoparticles according to a distribution of uptake rates, simulating a realistic uptake process. The specific uptake rate distribution was chosen to be log-normal, because our previous experimental data on polystyrene nanoparticle uptake by A549 cells is well-fitted by such a distribution [23,24]. Specifically, we used the same width of the distribution (σ = 0.5, where σ is the standard deviation of the corresponding normal distribution) and location (μ = 6.85, where μ is the mean of the corresponding normal distribution) that reproduces the experimental distributions (the location parameter is, however, less significant as our previous measurements were made in arbitrary fluorescence units). Upon cell division, the nanoparticles taken up were shared between the daughter cells with a given inheritance distribution [Eq (1)]. Time (*t*) is counted *after* the nanoparticle exposure. **A.** Distribution of number of nanoparticles per cell after an initial exposure for 0.17*T* amount of time and for symmetric inheritance [*p* = 0.5 in Eq (1)] and highly asymmetric inheritance (*p* = 0.8). Note the logarithmic abscissa axis, which suggests a very wide distribution in linear scale. **B.** Coefficient of variation of number of particles per cell, for cells exposed to nanoparticles for 0.17*T* amount of time, for different asymmetries of the inheritance distribution as indicated in the legend. **C-D.** The effect of the duration of nanoparticle exposure (as indicated in the legend) for **C.** symmetric inheritance [*p* = 0.5 in Eq (1)] and **D.** highly asymmetric inheritance (*p* = 0.8). Note that for panels C-D, the ordinate axis does not start at the origin to better show the time-evolution.

Having established the general idea for these simplified scenarios, we proceed by showing the results for a more realistic situation, more closely linked to potential experiments. Thus we consider a cell population that is exposed to, and take up, nanoparticles for a limited amount of time (a “pulse”) followed by a period during which the time-evolution of the population is subsequently followed (a “chase”). During the nanoparticle exposure, each cell takes up nanoparticles at a certain rate, which we assume remains fixed throughout the exposure. There are most likely random elements to the uptake process, which could limit the validity of assuming a fixed uptake rate. However, it is equally likely that there are also deterministic aspects to the uptake process, such as cell size [29], cell surface receptor expression and other cell characteristics that remain relatively fixed with time. Indeed, a model based on a fixed uptake rate agrees very well with the time evolution of the distribution of nanoparticles per cell measured experimentally in our previous work [23,24]. This empirical observation will be sufficient for our purposes here. The specific uptake rate distribution was chosen to be log-normal, because our previous experimental data on polystyrene nanoparticle uptake by A549 cells is well-fitted by such a distribution [23,24]. We thus used parameters that explicitly reproduce the experimental distributions from this work, though these measurements were made in terms of fluorescence (in arbitrary units) and so cannot readily be interpreted as actual particle numbers. Naturally, a log-normal uptake rate distribution produces at the end of the exposure a distribution of number of nanoparticles per cell that is also (roughly) log-normal (Fig 3A), a case which we have briefly already considered (S3 Fig). However, the uptake process coupled to cell division also produces a correlation between the age of a cell (the time since last division) and the number of nanoparticles it has taken up [23,24]. The reason is, loosely speaking, that recently divided (*i*.*e*., “young”) cells have just halved the number of nanoparticles taken up, while cells just about to divide (*i*.*e*., “old” cells) have had the longest time to take up nanoparticles without diluting their nanoparticles due to cell division. The correlation grows stronger the longer the cells are allowed to take up nanoparticles, and can be experimentally demonstrated by determining the number of nanoparticles in cells currently in a certain cell cycle phase [23]. We can also observe the effect in the distributions of number of nanoparticles per cell after the end of the exposure (Fig 3A). There, cell division leads to a tail at the lower end of the number of nanoparticles, something which is hardly visible for a symmetric inheritance distribution but is increasingly more evident for more asymmetric inheritance (*cf*. the black and green lines in Fig 3A). The correlation between cell age and number of nanoparticles will affect how the coefficient of variation evolves in time after a nanoparticle exposure. Furthermore, it introduces a dependence of how the coefficient of variation evolves in time on the length of the nanoparticle exposure.

We start by discussing the results for various asymmetries of the inheritance distribution with a fixed time of the nanoparticle exposure. We use a nanoparticle exposure of 0.17*T*, which corresponds to 4 h for a cell population doubling time of one day. This would seem to be a rather useful exposure duration: From a fundamental point of view, it is fairly short compared to the total length of one cell cycle, thus reducing the effect of the correlation between number of nanoparticles and cell age; conversely, from a practical point of view, it is still long enough to allow a sufficient number of nanoparticles to be taken up and hence give a strong enough signal. Fig 3B thus shows how the coefficient of variation evolves in time for the various asymmetries of the inheritance distribution. We may observe that for symmetric (or nearly symmetric) inheritance, there are oscillations in the coefficient of variation. Furthermore, while there is an overall increase (disregarding the local oscillations) in the coefficient of variation with time, it is very small and barely visible (it is more clear in Fig 3C below). For larger asymmetries, the oscillations are diminished and the overall growth becomes more pronounced. For highly asymmetric inheritance, the coefficient of variation grows essentially monotonically in time. All in all, the results are similar to the results we found without explicitly modelling the uptake process (S3 Fig). This is, of course, not surprising, given that we (intentionally) considered a fairly limited duration of nanoparticle exposure. Still, the effect of having an uptake that takes time *is* discernible, because the minima in the oscillations do not occur at full cell cycles (*t* = *T*, 2*T etc*) but rather are shifted towards earlier times.

To investigate the effect of the duration of the nanoparticle exposure in more detail, we compare how the coefficient of variation evolves with time for an exposure of 0.17*T*, 0.5*T* and *T* (corresponding to 4 h, 12 h and 24 h for a cell population doubling time of one day). Fig 3C shows the results for symmetric inheritance. The shorter nanoparticle exposure (0.17*T*) reproduces the results from Fig 3B, but due to the difference in scale, the oscillatory behaviour is more clear. For the medium exposure time (0.5*T*), the oscillations are also clear, but we additionally note a further shift in the time at which the minima occur. For the longer exposure time (*T*), the oscillations are barely visible, being washed out by the length of the nanoparticle exposure. Fig 3D shows the results for a highly asymmetric inheritance (*p* = 0.8). At this scale, the oscillations are somewhat visible for the shorter nanoparticle exposure (0.17*T*) but most likely too small to be observable by current experimental methodologies. For the longer nanoparticle exposures (0.5*T* and *T*) the oscillations are effectively gone. The results for a few other asymmetries of the inheritance distribution may be found in the Supporting Information (S4 Fig) which essentially shows a continuum between the two extremes discussed here.

At least at a qualitative level, these results are consistent with the few previous reports that have assessed the coefficient of variation experimentally. Thus, an early study that reported an asymmetric inheritance of 74% also reported an increasing, non-oscillatory, dependence of the coefficient of variation with time [13]. Furthermore, a more recent publication that reported an asymmetric inheritance of 64% also noted an increasing coefficient of variation (squared) [20].

To show the generality of the observations, we also performed simulations with other uptake rate distributions than those giving a good fit to our previous experimental data [23,24]. As already noted, using a (narrow) Gaussian distribution, we observe the same behaviour (S2 Fig). Furthermore, when using a log-normal distribution and varying the width of the distribution (S5 Fig) we observe different coefficients of variation in absolute terms, while when varying the position (S6 Fig) there is not much of an effect. In both cases, the *qualitative* trends, in particular the dependence on the asymmetry of the inheritance distribution, are identical to our previous observations (Fig 3). We also repeated the simulations with an uptake rate distribution chosen to agree with previous experimental observations on a different system, namely quantum dot uptake by A549 cells [29]. Again, the *quantitative* behaviour is different compared to Fig 3, but the *qualitative* behaviour is identical (S7 Fig).

Finally, we also relaxed the condition that all cells have the same total duration of their cell cycle. Thus, we assumed a distribution of cell cycle durations, with a coefficient of variation of 20% [30,31] and otherwise ran the simulations as above. Under these conditions, we observe oscillations in the coefficient of variation when the inheritance is symmetric (or nearly symmetric), while for larger asymmetries, the coefficient of variation grows and does so (nearly) monotonically (S8 Fig). In other words, the observations are qualitatively similar to what is observed when the total cell cycle duration is kept fixed (Fig 3), the main difference being that for symmetric inheritance, the overall increase of the coefficient of variation (upon which the oscillations are overlaid) is more pronounced. This is something that would be relevant only for fairly long experiments, as the effect is observable at timescales of several cell cycles, which amounts to several days of intermittent experimental observation for typical cell cycle durations.

**Fig 4:**
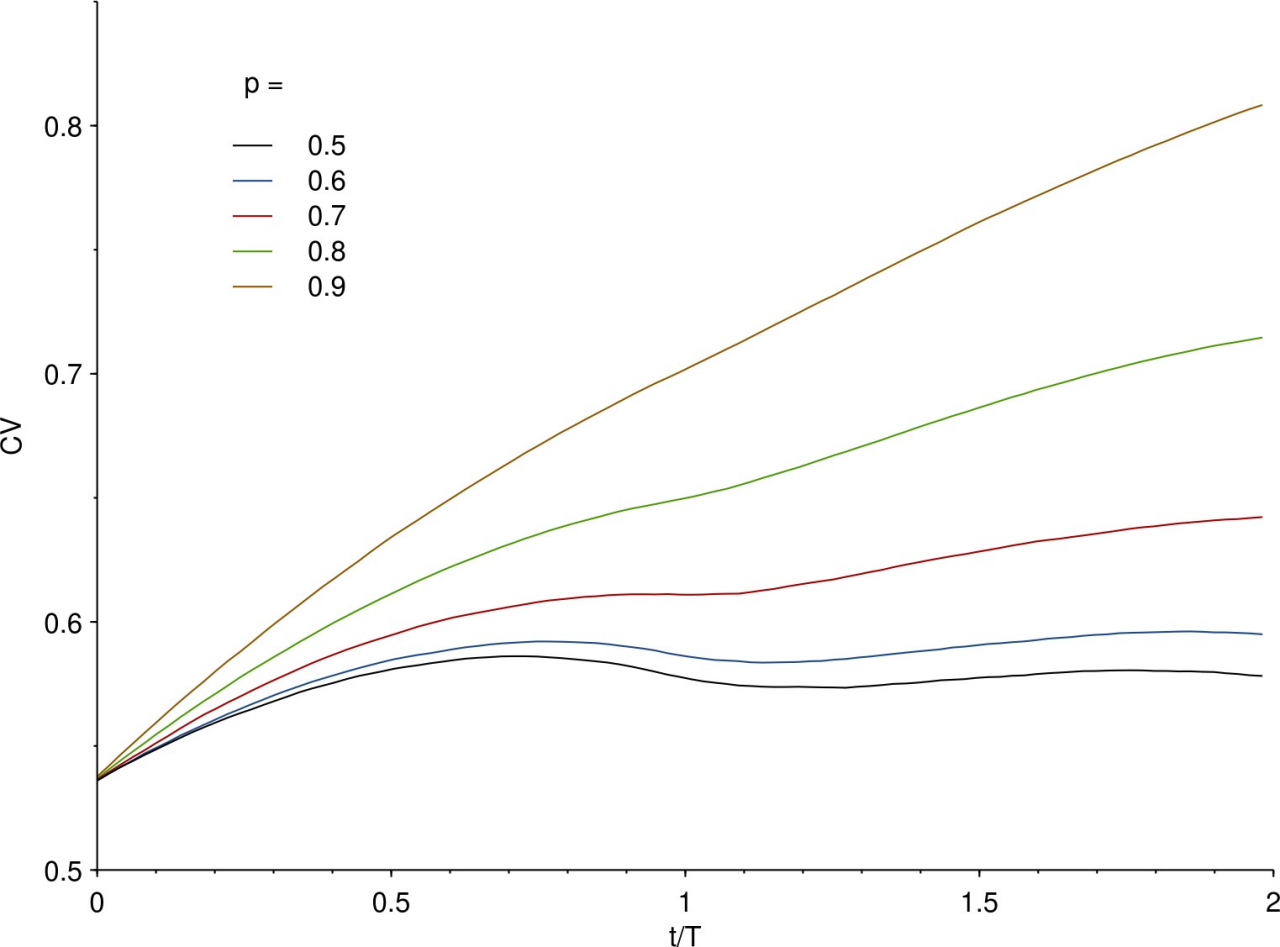
Time-evolution of the coefficient of variation of the number of nanoparticles per cell during continuous uptake. Cells were allowed to take up nanoparticles according to a distribution of uptake rates, simulating a realistic uptake process. The specific uptake rate distribution was chosen to be log-normal, because our previous experimental data on polystyrene nanoparticle uptake by A549 cells is well-fitted by such a distribution [23,24]. Specifically, we used the same width of the distribution (σ = 0.5, where σ is the standard deviation of the corresponding normal distribution) and location (μ = 6.85, where μ is the mean of the corresponding normal distribution) that reproduces the experimental distributions (the location parameter is, however, less significant as our previous measurements were made in arbitrary fluorescence units). Upon cell division, the nanoparticles taken up were shared between the daughter cells with a given inheritance distribution [Eq (1)]. The different lines represent the results for different asymmetries of the inheritance distribution, starting from a symmetric distribution [*p* = 0.5 in Eq (1)] towards a highly asymmetric one (increasing *p*) as indicated in the legend. Note that the ordinate axis does not start at the origin to better show the time-evolution.

For completeness, we also consider the experimental scenario that the cells are exposed continuously to nanoparticles and study how the coefficient of variation evolves in time under these circumstances. Again, we use a log-normal distribution of uptake rate of the cells, and specifically one that reproduces the experimental distributions from our previous experimental data on polystyrene nanoparticle uptake by A549 cells [23,24]. We also keep the assumption that the uptake rate is inherited by both daughters upon cell division. Fig 4 shows the results for various asymmetries of the inheritance distribution. We may observe that for a symmetric (or nearly symmetric) inheritance, the coefficient of variation exhibits a maximum before decreasing again, while a highly asymmetric inheritance is monotonically increasing. Thus, also under these (simulated) experimental conditions, one finds a rather strong dependence on asymmetry. While it would be difficult to discern the difference between a symmetric inheritance and, say, an asymmetry of *p* = 0.6, there is a much larger difference when compared to higher asymmetries (*cf*. the results for *p* = 0.7, 0.8 and 0.9). We expect such differences to be well within the range of what is experimentally observable.

## Conclusions

Previous efforts at demonstrating (potential) asymmetry in how nanoparticles are shared between daughter cells upon cell division have mainly focussed on the full distribution over cells, requiring either detailed modelling [13–16,18,20] or tedious experimental observations [19–22]. Here we have shown that the second moment over the statistical distribution, or more precisely the coefficient of variation, is sensitive to asymmetry and can be used for a more rapid read-out of the degree of asymmetry. This conclusion remains true for two common experimental scenarios, namely the evolution after a nanoparticle exposure limited in time and during continuous exposure, though the results look decidedly different.

After a concluded nanoparticle exposure, two features define how the coefficient of variation evolves in time: oscillations and an overall increase. For rather symmetric inheritance, the oscillations are prominent and hence provide the most sensitive test for the degree of asymmetry; conversely, for more asymmetric inheritance, the oscillations effectively disappear and instead an overall increase in the coefficient of variation will be the more sensitive observable to gauge the degree of asymmetry. To observe the oscillations, the nanoparticle exposure has to be as short as possible and the more asymmetric, the shorter the exposure is required.

During continuous uptake, instead, symmetric inheritance is characterised by a maximum of the coefficient of variation. The more asymmetric the inheritance, the less pronounced the maximum is and for high degrees of asymmetry, the coefficient of variation increases monotonically.

We expect that these observations are well within the range of what is observable with current experimental methodologies. We have not given explicit analytical expressions for how the coefficient of variation evolves with time that could be immediately fitted to data. Indeed, we expect such expressions to be more complex than useful. Nevertheless, the observations we present should be sufficient to allow a first, qualitative and easily accessible, assessment of the degree of asymmetry and thereby suggest if it is interesting to pursue these matters further with a more detailed study.

While we explicitly had high-throughput methods in mind for the experiments we simulate here, we note that the results could potentially also be used for comparing to experimental data measured by “medium-throughput” approaches. Thus, quantification of the mean over a distribution is inherently easier (*i*.*e*., fewer samples are needed) than the full distribution. From this point of view, the coefficient of variation (being essentially the second-order statistical descriptor, with the mean being the first) is certainly easier to sample well compared to the full distribution. Consequently, comparison to the results we show here may be possible already with a moderate number of cells.

We may also briefly comment on how this work fits into the wider context of “noise” (or variability) in biological systems [32], and that introduced by cell division in particular [28]. It is known that biomolecules [28] and organelles [33] can be partitioned stochastically and, particularly when the number of objects is low, this can lead to significant variability among cells (*cf*. Fig 1B and 1C). In the case of gene-expression specifically, this is well-studied and both experiment [34] and modelling [35–38] have made significant progress, leading to fully analytical results [36–38] now being available for direct comparison to experiments. A key difference with such studies is that here we consider exogenous objects (nanoparticles), rather than endogenous ones (biomolecules or organelles). The reason this is important is that cells have a mechanism for regenerating endogenous objects, while this is not relevant for exogenous objects (typically [39]). The models consequently differ in whether a “production process” has to be included or not. It has the further consequence that, say, the variability in gene expression can be quantified in terms of a time-independent (steady-state) variability [36,37]. In contrast, we have phrased our results in terms of the time-dependence of the coefficient of variation and, indeed, the lack of regeneration is the reason the time-dependence is interesting in the first place. Overall, we may view the present work as a generalisation of the variability introduced by cell division to account for the particularities of exogenous objects.

## Methods

Simulations were performed, where each cell was given an “age”, representing how far along its cell cycle it is. In the majority of cases, we assumed that the total duration of the cell cycle was the same for all cells and also that all cells progress along the cell cycle (“age”) at the same rate. As time progressed in the simulations, each cell was aged and the cells that reached the end of their cell cycle were started anew, but now as two cells. In order to simulate an exponentially growing cell population, we used an exponentially decaying initial age distribution where the number of cells at the very beginning of the cell cycle was twice as many as the number of cells at the very end. This also ensures that the age distribution does not change in time [23,24,26,40].

We also performed simulations where the total cell cycle duration was not the same for all cells (S8 Fig). We used an Erlang distribution of cell cycle durations, because it provides a reasonable fit to measured cell cycle durations reported in the literature [41]. Specifically, we used a distribution with a coefficient of variation set to 20%, corresponding to reported variations in the total cell cycle duration for cell lines [30,31], and a mean that gives the same cell population growth as when the cell cycle duration was held fixed. When a cell divided, the daughter cells were each assigned a random cell cycle duration from the same distribution. We initiated the simulations by positioning the cells along the cell cycle at random according to a uniform distribution, ensuring that cells were not started at a position beyond the total duration of their cell cycle. Subsequently we ran the simulation for several (mean) cell cycles, until the age distribution no longer changed, ensuring that the cell population grows exponentially and asynchronously. The consequent parts of the simulations were performed in the same way as when the total cell cycle duration was held fixed.

Each cell also contained a number of nanoparticles. When a cell reached the end of the cell cycle, a random number was taken based on the relevant inheritance distribution and one of the daughter cells was given this number of nanoparticles, while the other daughter cell was given the rest of the nanoparticles. We let the number of nanoparticles be a real (rather than natural) number, for simplicity and because when simulating the uptake process with a log-normal distribution of uptake rates, cells end up taking up a real number of nanoparticles. Consequently we also used a normal distribution approximation to the binomials in the inheritance distribution, because it thereby returns a real number. Thus, the inheritance distribution reads

$$\frac{1}{2}(N(np,np(1-p))+N(n(1-p),np(1-p)))$$ (2)

where *N*(μ, σ^2^) is a normal distribution of mean μ and standard deviation σ. This approximation is excellent for the kind of numbers we are considering here (see solid lines in Fig 1A) and does not have any effect on the results. Since the inheritance distribution in Eq (2) can occasionally result in a negative number of nanoparticles, we further used a cut-off so that only positive numbers were returned; again, these adjustments have no bearing on the final results.

For the simplest cases where we did not take into account nanoparticle uptake (Fig 2 and S1–S3 Figs) we simply initialised the cells with the stated starting distribution of number of nanoparticles, with no correlation between number of nanoparticles and cell age. For the more realistic scenarios (Figs 3 and 4 and S4–S8 Figs), the uptake process was also simulated. In this case, each cell was, before starting the simulation, given an uptake rate, *J*, from the stated distribution of uptake rates. Subsequently, each cell took up nanoparticles with this rate, such that in a time Δt, a cell would accumulate *J*Δt nanoparticles. Upon cell division, the accumulated nanoparticles were shared between daughter cells as described above. Furthermore, the uptake rate of the mother was also inherited by both daughter cells. When simulating the further evolution after a concluded nanoparticle exposure (Fig 3 and S4–S8 Figs), further uptake was simply ignored.

See also our previous work for further details, arguments and agreements between the results of the simulations and experiments [23–26].

## Supporting information

S1 Fig Time-evolution of the standard deviation of the number of nanoparticles per cell due to cell division with a narrow initial distribution. Upon cell division, the nanoparticles taken up were shared between the daughter cells with a given inheritance distribution [Eq (1)]. The different lines represent the results for different asymmetries of the inheritance distribution, starting from a symmetric distribution [*p* = 0.5 in Eq (1)] towards a highly asymmetric one (increasing *p*) as indicated in the legend. The initial distribution was chosen as a normal distribution with a standard deviation of 1. The results show that regardless of asymmetry (or, indeed, symmetry) the standard deviation always exhibits a maximum within the first cell cycle before reaching a second minimum after one full cell cycle. The results for *p* = 0.5 and *p* = 0.8 correspond to the coefficient of variation shown in Fig 2E and 2J, respectively.
(TIF)

S2 Fig Time-evolution of the coefficient of variation of the number of nanoparticles per cell due to cell division for different (initial) number of nanoparticles and a narrow initial distribution. Upon cell division, the nanoparticles taken up were shared between the daughter cells with a given inheritance distribution [Eq (1)]. The different lines represent the results for different asymmetries of the inheritance distribution, starting from a symmetric distribution [*p* = 0.5 in Eq (1)] towards a highly asymmetric one (increasing *p*) as indicated in the legend. The initial (mean) number of nanoparticles is indicated by the linestyle (solid, dashed, dotted; also indicated in the legend). The initial distribution was chosen as a normal distribution with a standard deviation of 1. The results show that regardless of the initial number of nanoparticles, the time-evolution of the coefficient of variation is roughly the same: For symmetric inheritance minor differences can be discerned, though they are likely too small to be measurable with current experimental methodologies; for more asymmetric inheritance, the differences are hardly visible at all.
(TIF)

S3 Fig Time-evolution of the coefficient of variation of the number of nanoparticles per cell due to cell division with a log-normal initial distribution. **A.** Choice of initial distribution of number of nanoparticles per cell. Note the logarithmic abscissa axis, which suggests a very wide distribution in linear scale. The initial distribution was chosen to be a log-normal distribution, because our previous experimental data on polystyrene nanoparticle uptake by A549 cells is well-fitted by such a distribution [23,24]. Specifically, we used a width of the distribution corresponding to the experimental one (σ = 0.5, where σ is the standard deviation of the corresponding normal distribution); the location of the distribution was fairly arbitrary, because the experimental data is in fluorescence rather than particle numbers (μ = ln 100, where μ is the mean of the corresponding normal distribution). **B.** Upon cell division, the nanoparticles taken up were shared between the daughter cells with a given inheritance distribution [Eq (1)]. The different lines represent the results for different asymmetries of the inheritance distribution, starting from a symmetric distribution [*p* = 0.5 in Eq (1)] towards a highly asymmetric one (increasing *p*) as indicated in the legend. The results are rather similar compared to the situation where the initial distribution is a narrow normal distribution (Fig 2E and 2J and S2 Fig) in that the coefficient of variation exhibits clear oscillations for symmetric or nearly symmetric inheritance, but becomes almost monotonically increasing the larger the asymmetry of the inheritance. A difference is that the starting (*t* = 0) coefficient of variation is distinctly larger for a log-normal distribution compared to the narrow normal distribution (Fig 2 and S2 Fig).
(TIF)

S4 Fig Time-evolution of the coefficient of variation of the number of nanoparticles per cell after a nanoparticle exposure. Cells were allowed to take up nanoparticles for a limited period of time (“pulse”) of duration 0.17*T*, 0.5*T* and *T* (indicated in the legends) and then followed (“chased”). During the exposure, the cells took up nanoparticles according to a distribution of uptake rates, simulating a realistic uptake process. The specific uptake rate distribution was chosen to be log-normal, because our previous experimental data on polystyrene nanoparticle uptake by A549 cells is well-fitted by such a distribution [23,24]. Specifically, we used the same width of the distribution (σ = 0.5, where σ is the standard deviation of the corresponding normal distribution) and location (μ = 6.85, where μ is the mean of the corresponding normal distribution) that reproduces the experimental distributions (the location parameter is, however, less significant as our previous measurements were made in arbitrary fluorescence units). Upon cell division, the nanoparticles taken up were shared between the daughter cells with a given inheritance distribution [Eq (1)]. Time (*t*) is counted *after* the nanoparticle exposure. The different panels show the results for different asymmetries of the inheritance distribution. **A.** *p* = 0.6; **B.** *p* = 0.7; **C.** *p* = 0.9. The results for symmetric inheritance (*p* = 0.5) and *p* = 0.8 may be found in Fig 3C and 3D. Note that the ordinate axis does not start at the origin to better show the time-evolution.
(TIF)

S5 Fig Dependence of the time-evolution of the coefficient of variation of the number of nanoparticles per cell after a nanoparticle exposure on the width of the uptake rate distribution. Cells were allowed to take up nanoparticles for a limited period of time (“pulse”) and then followed (“chased”). During the exposure, the cells took up nanoparticles according to a distribution of uptake rates, simulating a realistic uptake process. The specific uptake rate distribution was chosen to be log-normal, because our previous experimental data on polystyrene nanoparticle uptake by A549 cells is well-fitted by such a distribution [23,24]. Specifically, we used the same location of the distribution (μ = 6.85, where μ is the mean of the corresponding normal distribution) that reproduces the experimental distributions. The width of the distribution (in terms of σ, the standard deviation of the corresponding normal distribution) was varied, both making it more narrow (σ = 0.25) and wider (σ = 0.75) than that reproducing the experimental distributions (σ = 0.50). Upon cell division, the nanoparticles taken up were shared between the daughter cells with a given inheritance distribution [Eq (1)]. Time (*t*) is counted *after* the nanoparticle exposure. (Rows) Variation with the symmetry of the inheritance distribution, ranging from completely symmetric inheritance [*p* = 0.5 in Eq (1)] to highly asymmetric inheritance (*p* = 0.9). (Left column) Coefficient of variation in absolute terms. (Right column) Coefficient of variation “normalised” by subtraction of the mean value. The results are in qualitative agreement with those simulating experimental systems (Fig 3 and S7 Fig below) as well as when varying the location of the uptake rate distribution (S6 Fig below) demonstrating the generality of the observations.
(TIF)

S6 Fig Dependence of the time-evolution of the coefficient of variation of the number of nanoparticles per cell after a nanoparticle exposure on the location of the uptake rate distribution. Cells were allowed to take up nanoparticles for a limited period of time (“pulse”) and then followed (“chased”). During the exposure, the cells took up nanoparticles according to a distribution of uptake rates, simulating a realistic uptake process. The specific uptake rate distribution was chosen to be log-normal, because our previous experimental data on polystyrene nanoparticle uptake by A549 cells is well-fitted by such a distribution [23,24]. Specifically, we used the same width of the distribution (σ = 0.5, where σ is the standard deviation of the corresponding normal distribution) that reproduces the experimental distributions. The location of the distribution (in terms of μ, the mean of the corresponding normal distribution) was varied (μ = 5, 7, 9, where μ = 6.85 is the value that reproduces the experimental distributions). Upon cell division, the nanoparticles taken up were shared between the daughter cells with a given inheritance distribution [Eq (1)]. (Rows) Variation with the symmetry of the inheritance distribution, ranging from completely symmetric inheritance [*p* = 0.5 in Eq (1)] to highly asymmetric inheritance (*p* = 0.9). **A-E.** Coefficient of variation as a function of time, where time (*t*) is counted *after* the nanoparticle exposure. The results are in qualitative agreement with those simulating experimental systems (Fig 3 and S7 Fig below) as well as when varying the width of the uptake rate distribution (S5 Fig) demonstrating the generality of the observations.
(TIF)

S7 Fig Time-evolution after a nanoparticle exposure simulating another system that has been studied experimentally. Previous work has quantified the distribution of number of nanoparticles per cell (technically, the number of nanoparticle-containing vesicles) for A549 cells exposed to quantum dots and developed a theoretical model to describe it [29]. We can thereby use this data/model as another example where to test our approach. Thus, cells were allowed to take up nanoparticles for a limited period of time (“pulse”) and then followed (“chased”). During the exposure, the cells took up nanoparticles according to a log-normal distribution of uptake rates with parameters (σ = 0.55 and μ = 5.2, where σ is the standard deviation and μ is the mean, respectively, of the corresponding normal distribution) such that it fits the model describing quantum dot uptake by A549 cells [29]. Upon cell division, the nanoparticles taken up were shared between the daughter cells with a given inheritance distribution (Eq 1). The cell population doubling time was set to 22 h, as in our previous work on the same (A549) cells [23]. **A.** Distribution of number of nanoparticles per cell after the initial exposure for 4 h and for symmetric inheritance [*p* = 0.5 in Eq (1)]. (Solid line) Results of the previously developed model [29], with parameters chosen to correspond to A549 cells, a quantum dot concentration of 4 nM and a 4 h exposure time. (Dotted line) Log-normal approximation of the exact model. Note that the log-normal distribution approximation has a slightly fatter tail than the exact model, but since the exact model often somewhat underestimates the number of cells with high particle numbers observed experimentally [29], we consider this a feature. **B.** Coefficient of variation as a function of time for different asymmetries of the inheritance distribution as indicated in the legend. Time (*t*) is counted *after* the nanoparticle exposure. The results are in qualitative agreement with those simulating another experimental system (Fig 3) as well as when varying the width (S5 Fig) and location (S6 Fig) of the uptake rate distribution, demonstrating the generality of the observations.
(TIF)

S8 Fig Time-evolution after a nanoparticle exposure with a distribution of total cell cycle duration. Rather than a fixed total cell cycle duration, cells were assigned a cell cycle duration from an Erlang distribution with a coefficient of variation of 20%. We use this particular distribution because it provides a reasonable fit to measured cell cycle durations reported in the literature [41]. When a cell divided, the daughter cells were each assigned a random cell cycle duration from the same distribution. Cells were allowed to take up nanoparticles for a limited period of time (“pulse”), 0.17*T*, and then followed (“chased”). During the exposure, the cells took up nanoparticles according to a distribution of uptake rates, simulating a realistic uptake process. The specific uptake rate distribution was chosen to be log-normal, because our previous experimental data on polystyrene nanoparticle uptake by A549 cells is well-fitted by such a distribution [23,24]. Specifically, we used the same width of the distribution (σ = 0.5, where σ is the standard deviation of the corresponding normal distribution) and location (μ = 6.85, where μ is the mean of the corresponding normal distribution) that reproduces the experimental distributions (the location parameter is, however, less significant as our previous measurements were made in arbitrary fluorescence units). Upon cell division, the nanoparticles taken up were shared between the daughter cells with a given inheritance distribution [Eq (1)]. **A.** Coefficient of variation as a function of time for different asymmetries of the inheritance distribution, as indicated in the legend. **B-F.** Same results shown individually, so as to better show the variation. Note that for this reason, the ordinate axis does not start at the origin and the axes are different for the different panels. Time (*t*) is counted *after* the nanoparticle exposure. The results are in qualitative agreement with those where the total cell cycle duration was held fixed (Fig 3), the main difference being that for symmetric inheritance (panel B) the overall increase observed over multiple cell cycles is more pronounced.
(TIF)
