# Supplementary figures and images for: Asymmetry of nanoparticle inheritance upon cell division: Effect on the coefficient of variation
Source: PLoS One. 2020 Nov 17;15(11):e0242547. doi: 10.1371/journal.pone.0242547 (PMC7671523; doi:10.1371/journal.pone.0242547)

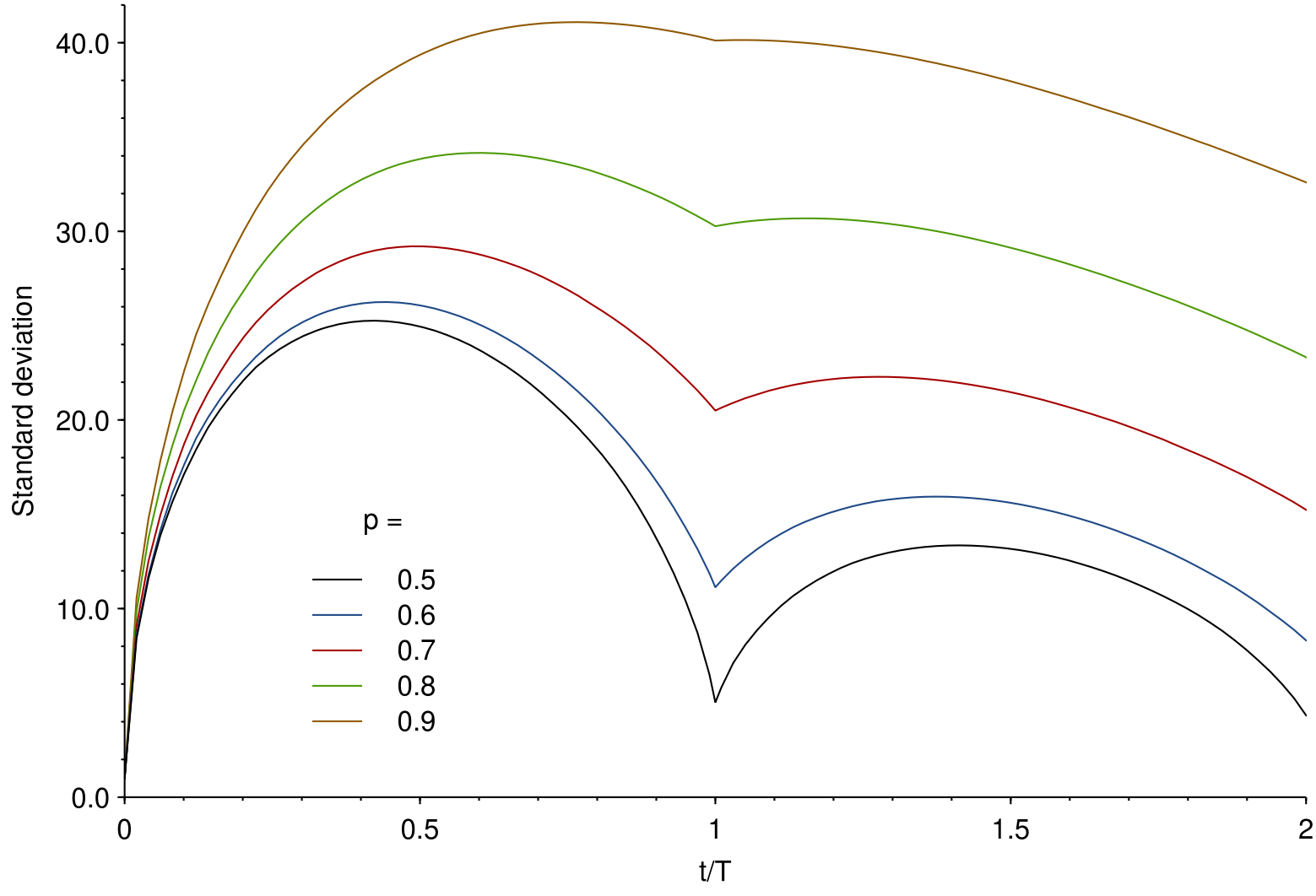

Supplement: S1 Fig — Upon cell division, the nanoparticles taken up were shared between the daughter cells with a given inheritance distribution [Eq (1)]. The different lines represent the results for different asymmetries of the inheritance distribution, starting from a symmetric distribution [p = 0.5 in Eq (1)] towards a highly asymmetric one (increasing p) as indicated in the legend. The initial distribution was chosen as a normal distribution with a standard deviation of 1. The results show that regardless of asymmetry (or, indeed, symmetry) the standard deviation always exhibits a maximum within the first cell cycle before reaching a second minimum after one full cell cycle. The results for p = 0.5 and p = 0.8 correspond to the coefficient of variation shown in Fig 2E and 2J, respectively. (TIF) [file pone.0242547.s001.tif]

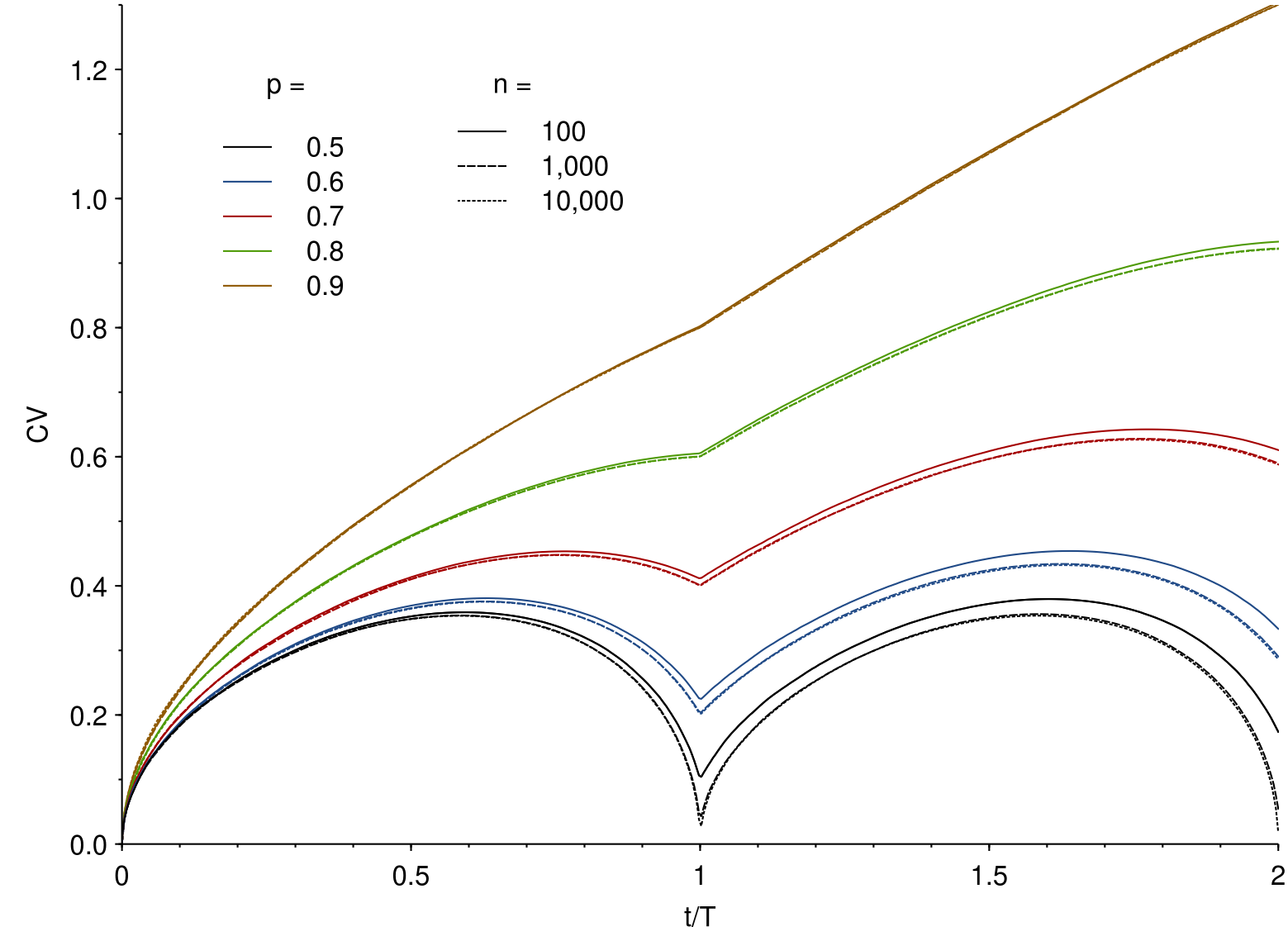

Supplement: S2 Fig — Upon cell division, the nanoparticles taken up were shared between the daughter cells with a given inheritance distribution [Eq (1)]. The different lines represent the results for different asymmetries of the inheritance distribution, starting from a symmetric distribution [p = 0.5 in Eq (1)] towards a highly asymmetric one (increasing p) as indicated in the legend. The initial (mean) number of nanoparticles is indicated by the linestyle (solid, dashed, dotted; also indicated in the legend). The initial distribution was chosen as a normal distribution with a standard deviation of 1. The results show that regardless of the initial number of nanoparticles, the time-evolution of the coefficient of variation is roughly the same: For symmetric inheritance minor differences can be discerned, though they are likely too small to be measurable with current experimental methodologies; for more asymmetric inheritance, the differences are hardly visible at all. (TIF) [file pone.0242547.s002.tif]

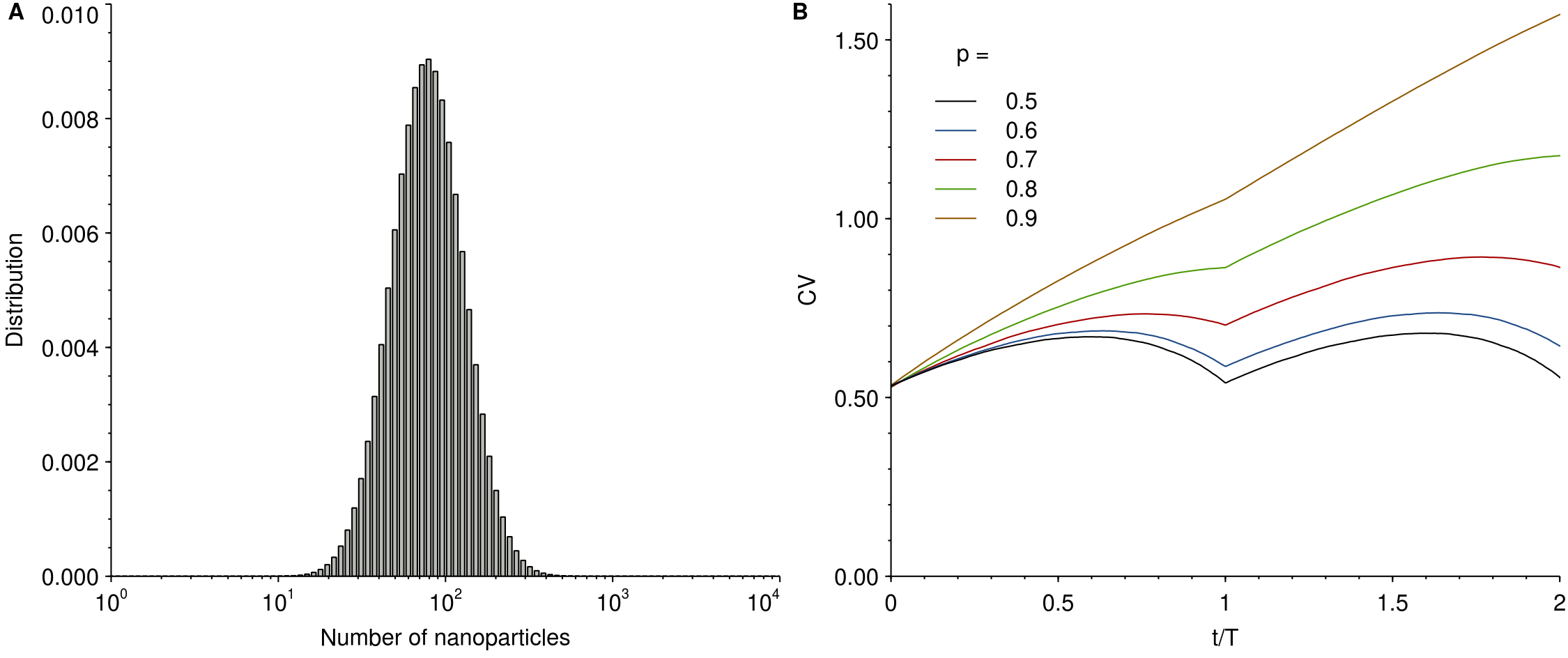

Supplement: S3 Fig — A. Choice of initial distribution of number of nanoparticles per cell. Note the logarithmic abscissa axis, which suggests a very wide distribution in linear scale. The initial distribution was chosen to be a log-normal distribution, because our previous experimental data on polystyrene nanoparticle uptake by A549 cells is well-fitted by such a distribution [23,24]. Specifically, we used a width of the distribution corresponding to the experimental one (σ = 0.5, where σ is the standard deviation of the corresponding normal distribution); the location of the distribution was fairly arbitrary, because the experimental data is in fluorescence rather than particle numbers (μ = ln 100, where μ is the mean of the corresponding normal distribution). B. Upon cell division, the nanoparticles taken up were shared between the daughter cells with a given inheritance distribution [Eq (1)]. The different lines represent the results for different asymmetries of the inheritance distribution, starting from a symmetric distribution [p = 0.5 in Eq (1)] towards a highly asymmetric one (increasing p) as indicated in the legend. The results are rather similar compared to the situation where the initial distribution is a narrow normal distribution (Fig 2E and 2J and S2 Fig) in that the coefficient of variation exhibits clear oscillations for symmetric or nearly symmetric inheritance, but becomes almost monotonically increasing the larger the asymmetry of the inheritance. A difference is that the starting (t = 0) coefficient of variation is distinctly larger for a log-normal distribution compared to the narrow normal distribution (Fig 2 and S2 Fig). (TIF) [file pone.0242547.s003.tif]

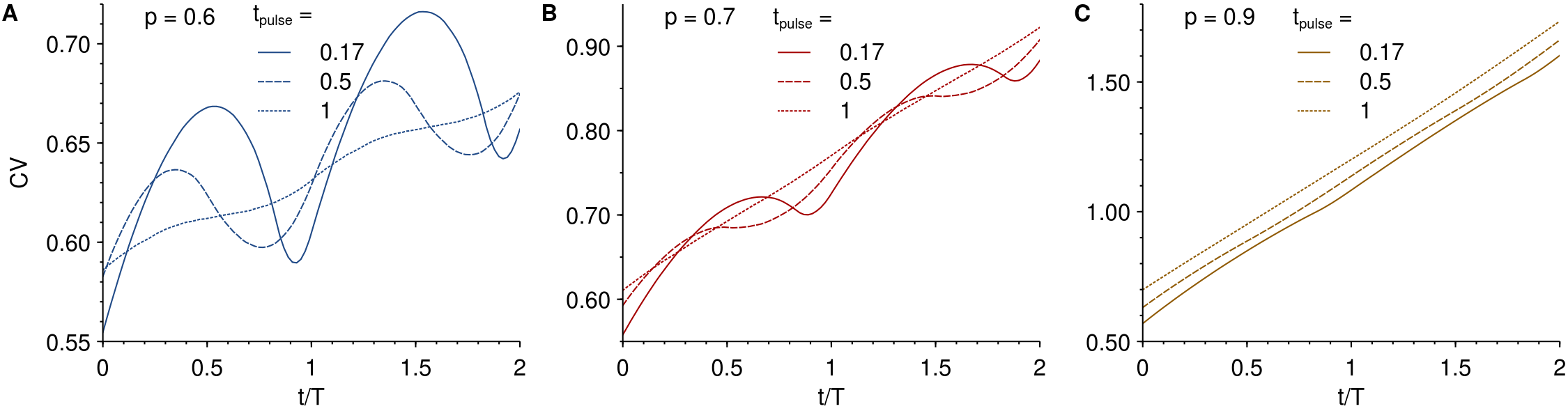

Supplement: S4 Fig — Cells were allowed to take up nanoparticles for a limited period of time (“pulse”) of duration 0.17T, 0.5T and T (indicated in the legends) and then followed (“chased”). During the exposure, the cells took up nanoparticles according to a distribution of uptake rates, simulating a realistic uptake process. The specific uptake rate distribution was chosen to be log-normal, because our previous experimental data on polystyrene nanoparticle uptake by A549 cells is well-fitted by such a distribution [23,24]. Specifically, we used the same width of the distribution (σ = 0.5, where σ is the standard deviation of the corresponding normal distribution) and location (μ = 6.85, where μ is the mean of the corresponding normal distribution) that reproduces the experimental distributions (the location parameter is, however, less significant as our previous measurements were made in arbitrary fluorescence units). Upon cell division, the nanoparticles taken up were shared between the daughter cells with a given inheritance distribution [Eq (1)]. Time (t) is counted after the nanoparticle exposure. The different panels show the results for different asymmetries of the inheritance distribution. A. p = 0.6; B. p = 0.7; C. p = 0.9. The results for symmetric inheritance (p = 0.5) and p = 0.8 may be found in Fig 3C and 3D. Note that the ordinate axis does not start at the origin to better show the time-evolution. (TIF) [file pone.0242547.s004.tif]

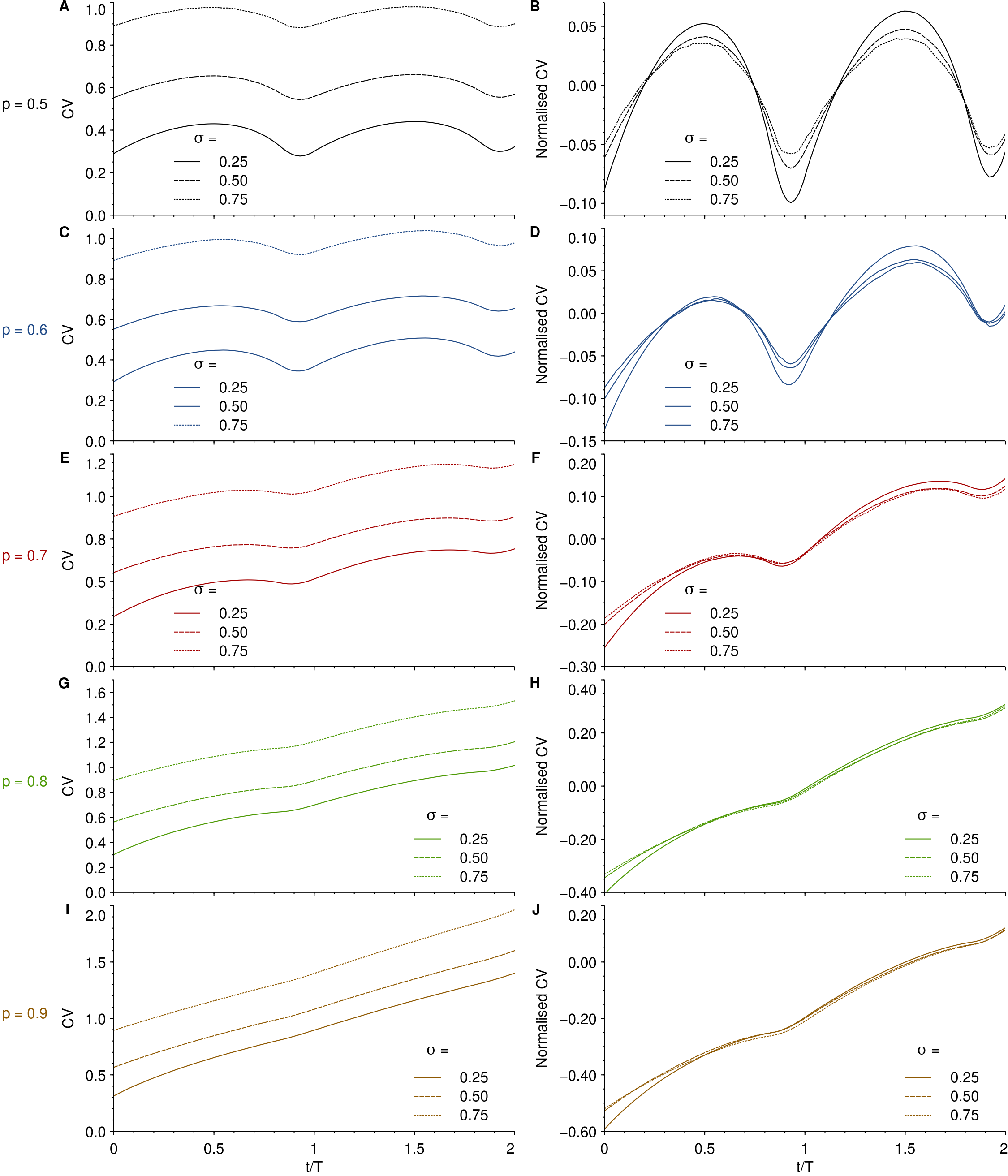

Supplement: S5 Fig — Cells were allowed to take up nanoparticles for a limited period of time (“pulse”) and then followed (“chased”). During the exposure, the cells took up nanoparticles according to a distribution of uptake rates, simulating a realistic uptake process. The specific uptake rate distribution was chosen to be log-normal, because our previous experimental data on polystyrene nanoparticle uptake by A549 cells is well-fitted by such a distribution [23,24]. Specifically, we used the same location of the distribution (μ = 6.85, where μ is the mean of the corresponding normal distribution) that reproduces the experimental distributions. The width of the distribution (in terms of σ, the standard deviation of the corresponding normal distribution) was varied, both making it more narrow (σ = 0.25) and wider (σ = 0.75) than that reproducing the experimental distributions (σ = 0.50). Upon cell division, the nanoparticles taken up were shared between the daughter cells with a given inheritance distribution [Eq (1)]. Time (t) is counted after the nanoparticle exposure. (Rows) Variation with the symmetry of the inheritance distribution, ranging from completely symmetric inheritance [p = 0.5 in Eq (1)] to highly asymmetric inheritance (p = 0.9). (Left column) Coefficient of variation in absolute terms. (Right column) Coefficient of variation “normalised” by subtraction of the mean value. The results are in qualitative agreement with those simulating experimental systems (Fig 3 and S7 Fig below) as well as when varying the location of the uptake rate distribution (S6 Fig below) demonstrating the generality of the observations. (TIF) [file pone.0242547.s005.tif]

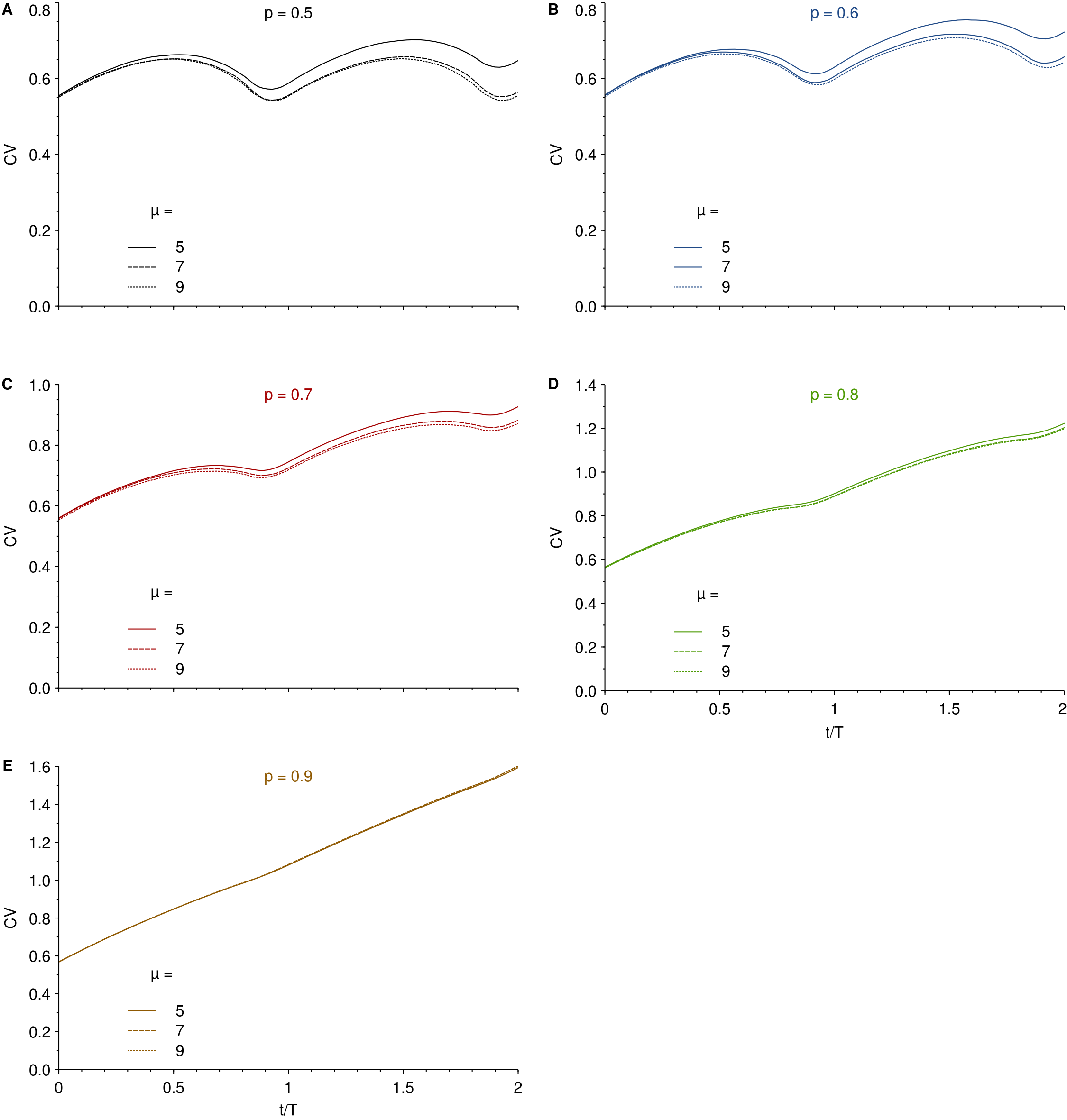

Supplement: S6 Fig — Cells were allowed to take up nanoparticles for a limited period of time (“pulse”) and then followed (“chased”). During the exposure, the cells took up nanoparticles according to a distribution of uptake rates, simulating a realistic uptake process. The specific uptake rate distribution was chosen to be log-normal, because our previous experimental data on polystyrene nanoparticle uptake by A549 cells is well-fitted by such a distribution [23,24]. Specifically, we used the same width of the distribution (σ = 0.5, where σ is the standard deviation of the corresponding normal distribution) that reproduces the experimental distributions. The location of the distribution (in terms of μ, the mean of the corresponding normal distribution) was varied (μ = 5, 7, 9, where μ = 6.85 is the value that reproduces the experimental distributions). Upon cell division, the nanoparticles taken up were shared between the daughter cells with a given inheritance distribution [Eq (1)]. (Rows) Variation with the symmetry of the inheritance distribution, ranging from completely symmetric inheritance [p = 0.5 in Eq (1)] to highly asymmetric inheritance (p = 0.9). A-E. Coefficient of variation as a function of time, where time (t) is counted after the nanoparticle exposure. The results are in qualitative agreement with those simulating experimental systems (Fig 3 and S7 Fig below) as well as when varying the width of the uptake rate distribution (S5 Fig) demonstrating the generality of the observations. (TIF) [file pone.0242547.s006.tif]

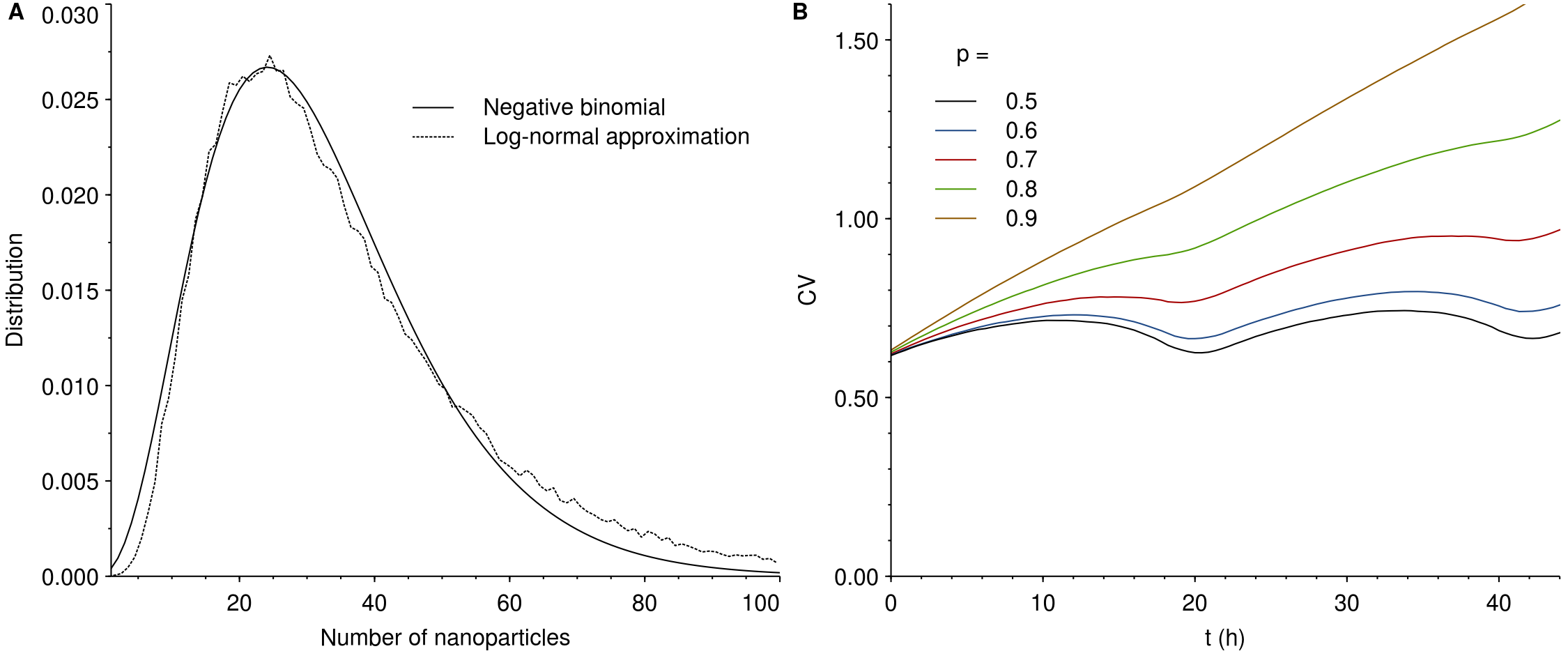

Supplement: S7 Fig — Previous work has quantified the distribution of number of nanoparticles per cell (technically, the number of nanoparticle-containing vesicles) for A549 cells exposed to quantum dots and developed a theoretical model to describe it [29]. We can thereby use this data/model as another example where to test our approach. Thus, cells were allowed to take up nanoparticles for a limited period of time (“pulse”) and then followed (“chased”). During the exposure, the cells took up nanoparticles according to a log-normal distribution of uptake rates with parameters (σ = 0.55 and μ = 5.2, where σ is the standard deviation and μ is the mean, respectively, of the corresponding normal distribution) such that it fits the model describing quantum dot uptake by A549 cells [29]. Upon cell division, the nanoparticles taken up were shared between the daughter cells with a given inheritance distribution (Eq 1). The cell population doubling time was set to 22 h, as in our previous work on the same (A549) cells [23]. A. Distribution of number of nanoparticles per cell after the initial exposure for 4 h and for symmetric inheritance [p = 0.5 in Eq (1)]. (Solid line) Results of the previously developed model [29], with parameters chosen to correspond to A549 cells, a quantum dot concentration of 4 nM and a 4 h exposure time. (Dotted line) Log-normal approximation of the exact model. Note that the log-normal distribution approximation has a slightly fatter tail than the exact model, but since the exact model often somewhat underestimates the number of cells with high particle numbers observed experimentally [29], we consider this a feature. B. Coefficient of variation as a function of time for different asymmetries of the inheritance distribution as indicated in the legend. Time (t) is counted after the nanoparticle exposure. The results are in qualitative agreement with those simulating another experimental system (Fig 3) as well as when varying the width (S5 Fig) and location (S6 Fig) of [file pone.0242547.s007.tif]

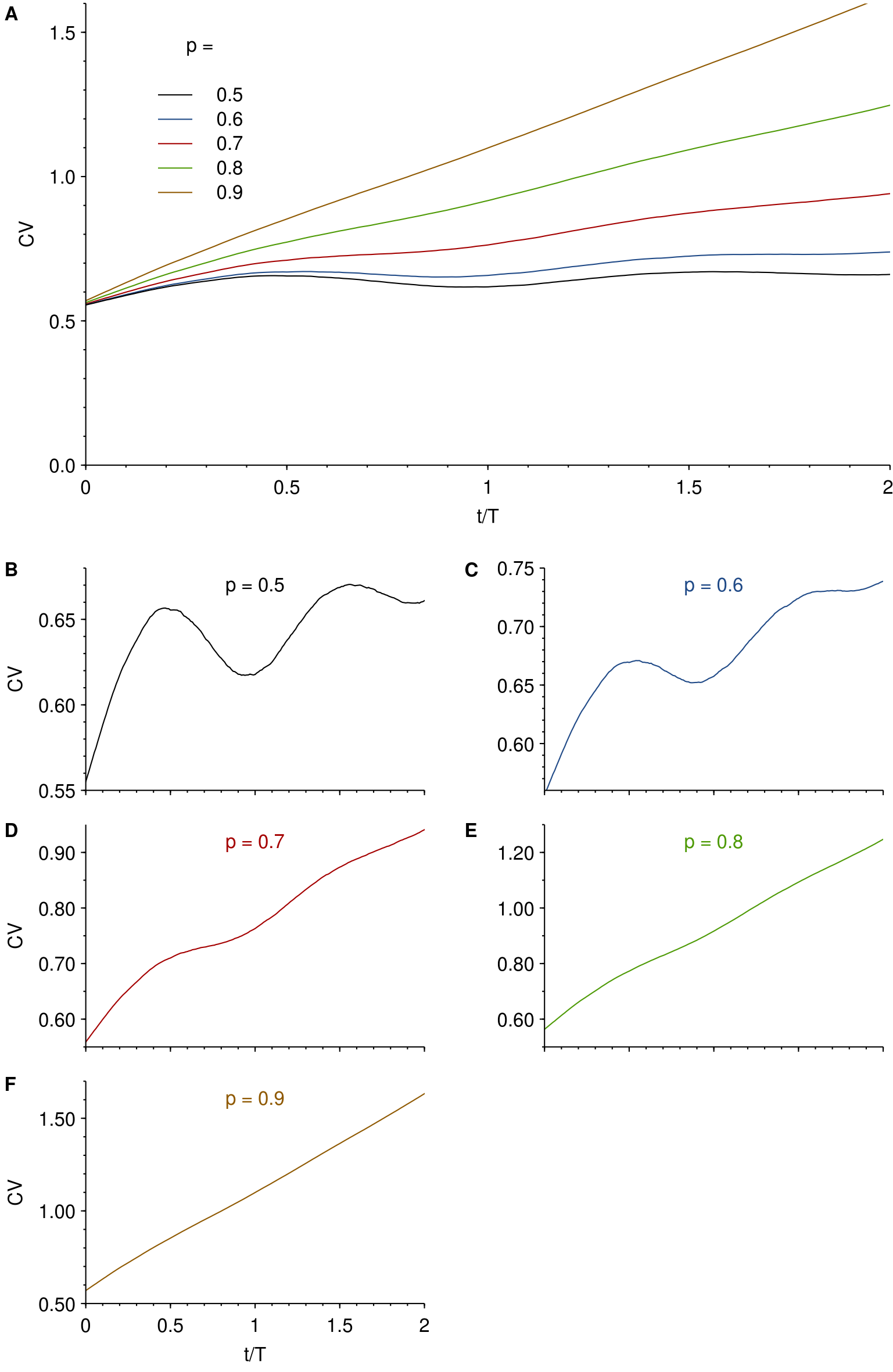

Supplement: S8 Fig — Rather than a fixed total cell cycle duration, cells were assigned a cell cycle duration from an Erlang distribution with a coefficient of variation of 20%. We use this particular distribution because it provides a reasonable fit to measured cell cycle durations reported in the literature [41]. When a cell divided, the daughter cells were each assigned a random cell cycle duration from the same distribution. Cells were allowed to take up nanoparticles for a limited period of time (“pulse”), 0.17T, and then followed (“chased”). During the exposure, the cells took up nanoparticles according to a distribution of uptake rates, simulating a realistic uptake process. The specific uptake rate distribution was chosen to be log-normal, because our previous experimental data on polystyrene nanoparticle uptake by A549 cells is well-fitted by such a distribution [23,24]. Specifically, we used the same width of the distribution (σ = 0.5, where σ is the standard deviation of the corresponding normal distribution) and location (μ = 6.85, where μ is the mean of the corresponding normal distribution) that reproduces the experimental distributions (the location parameter is, however, less significant as our previous measurements were made in arbitrary fluorescence units). Upon cell division, the nanoparticles taken up were shared between the daughter cells with a given inheritance distribution [Eq (1)]. A. Coefficient of variation as a function of time for different asymmetries of the inheritance distribution, as indicated in the legend. B-F. Same results shown individually, so as to better show the variation. Note that for this reason, the ordinate axis does not start at the origin and the axes are different for the different panels. Time (t) is counted after the nanoparticle exposure. The results are in qualitative agreement with those where the total cell cycle duration was held fixed (Fig 3), the main difference being that for symmetric inheritance (panel B) the overall increase [file pone.0242547.s008.tif]
